# Supplementary material for: TET2-mediated tumor cGAS triggers endothelial STING activation to regulate vasculature remodeling and anti-tumor immunity in liver cancer
Source: Nat Commun. 2024 Jan 4;15:6. doi: 10.1038/s41467-023-43743-9 (PMC10766952; doi:10.1038/s41467-023-43743-9)
Supplement: Supplementary file 1 — Supplementary information [file 41467_2023_43743_MOESM1_ESM.pdf]

Supplementary Information

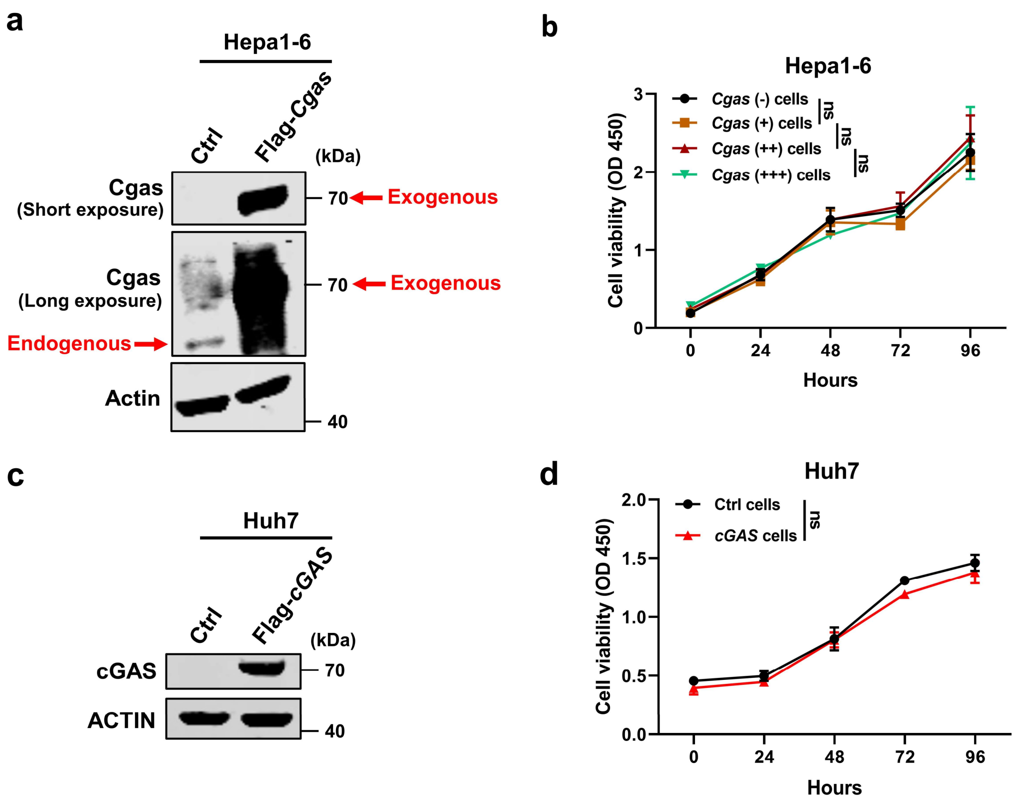

Supplementary Fig. 1 cGAS overexpression does not alter liver cancer cell proliferation. a Cgas protein level in Hepa1-6 cells with *Cgas* overexpression (Hepa1-6-*Cgas*) or the corresponding Ctrl cells (Hepa1-6-Ctrl). **b** Proliferation curves of Hepa1-6 cells with *Cgas* overexpression (n = 4). **c** cGAS protein level in Huh7 cells with *cGAS* overexpression (Huh7-*cGAS*) or the corresponding Ctrl cells (Huh7-Ctrl). **d** Proliferation curves of Huh7-*cGAS* cells versus Ctrl cells (n = 4). Representative of n = 3 independent experiments (**a**, **c**). *P* values are calculated using two-way ANOVA (**b**, **d**). ns, not significant. Source data are provided as a Source Data file.

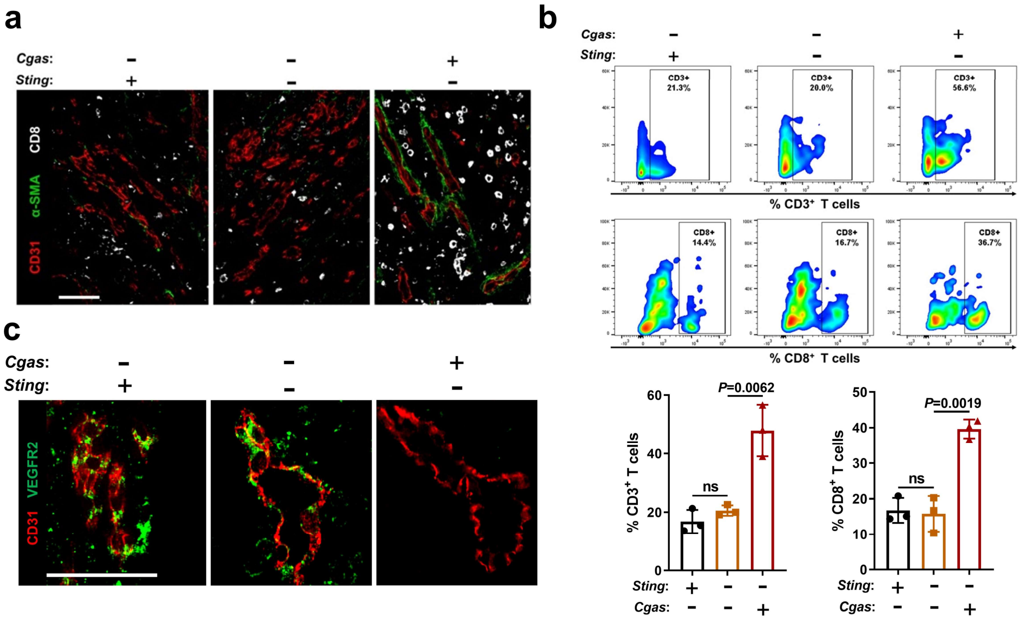

Supplementary Fig. 2 Tumor cGAS regulates vascular normalization, maturation, and immune cell infiltration in an intrinsic STING-independent manner. a Representative immunofluorescence images for pericyte (α-SMA^{+}) coverage of tumor vessels (CD31^{+}) and CD8^{+} T cells in indicated tumors. Scale bars, 100 µm. **b** Flow cytometric analysis and frequency of CD3^{+} T cells and CD8^{+} T cells in indicated tumors (n = 3). **c** Representative immunofluorescence images for VEGFR2^{+} tumor vessels (CD31^{+}) in indicated tumors. Scale bars, 50 µm. Representative of n = 3 independent experiments (**a**, **c**). *P* values are calculated using one-way ANOVA (**b**). ns, not significant. Source data are provided as a Source Data file.

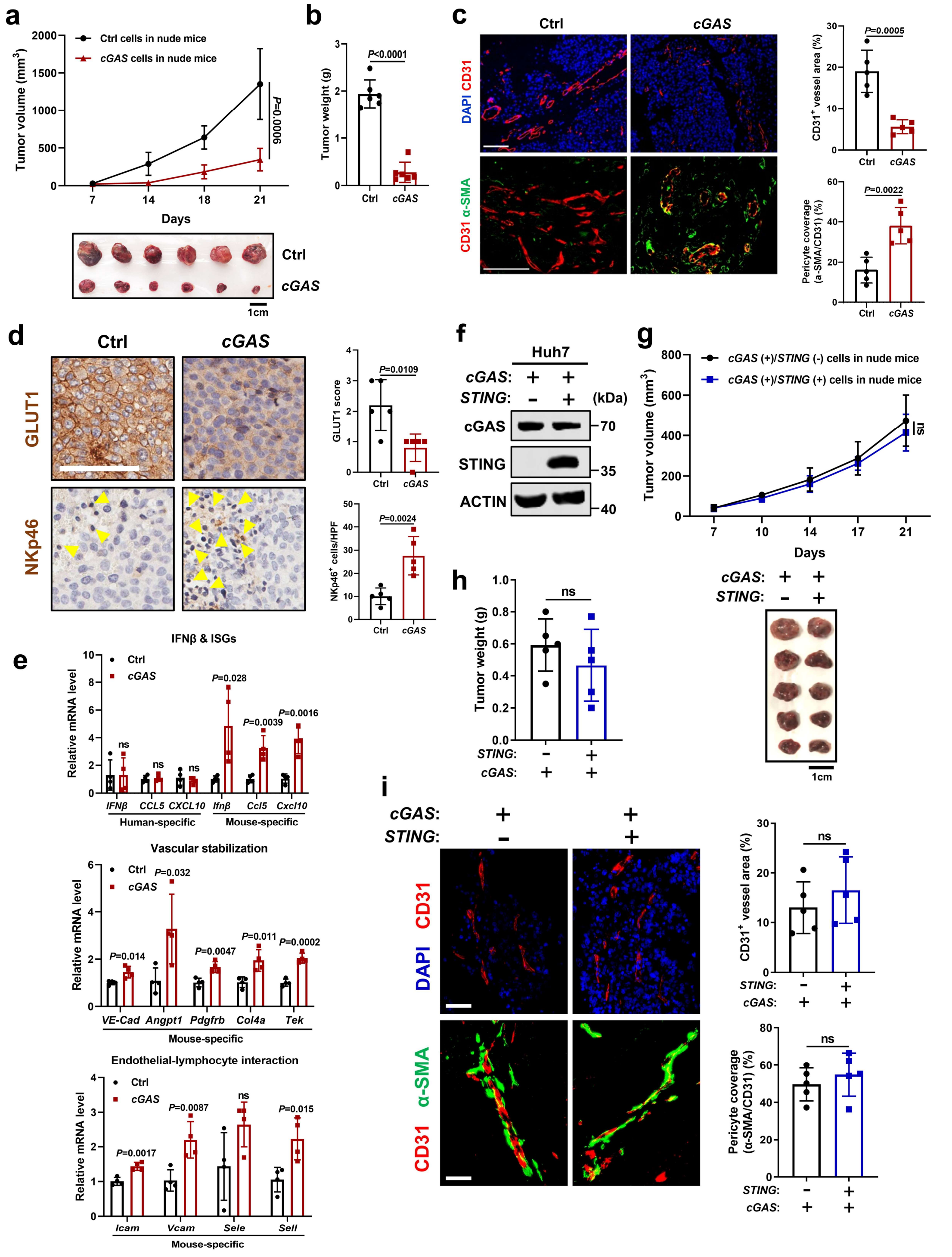

Supplementary Fig. 3 Human liver cancer cGAS drives host STING activation, vascular normalization, and tumor repression. a, **b** Tumor growth curves (**a**) and tumor burdens (**b**) in nude mice injected subcutaneously with Huh7-*cGAS* or Ctrl cells for 3 weeks (n = 6 mice per group). **c** Representative immunofluorescence images and quantification for CD31^{+} vessels density and pericyte (α-SMA^{+}) coverage of tumor vessels (CD31^{+}) in Huh7-*cGAS* or Ctrl cells-derived tumors (n = 5). Scale bars, 100 µm. **d** Representative immunohistochemical images and quantification for GLUT1^{+} hypoxic area and NK cells (NKp46^{+}) in Huh7-*cGAS* or Ctrl cells-

derived tumors (n = 5). The yellow arrows represent NKp46$^+$ cells. Scale bars, 100 µm. **e** mRNA levels of human-specific and mouse-specific genes related to IFNβ and ISGs, vascular stabilization, and endothelial-lymphocyte interaction in Huh7-*cGAS* or Ctrl cells-derived tumors (n = 4). **f** cGAS and STING protein levels in Huh7 cells with *cGAS* and *STING* overexpression. Representative of n = 3 independent experiments. **g**, **h** Tumor growth curves (**g**) and tumor burdens (**h**) in nude mice injected subcutaneously with Huh7-*cGAS* (+)/*STING* (+) or *cGAS* (+)/*STING* (-) cells for 3 weeks (n = 5 mice per group). **i** Representative immunofluorescence images and quantification for CD31$^+$ vessels density and pericyte (α-SMA$^+$) coverage of tumor vessels (CD31$^+$) in Huh7-*cGAS* (+)/*STING* (+) or *cGAS* (+)/*STING* (-) cells-derived tumors (n = 5). Scale bars, 50 µm. *P* values are calculated using two-way ANOVA (**a**, **g**) and two-tailed unpaired Student's *t* test (**b-e**, **h**, **i**). ns, not significant. Source data are provided as a Source Data file.

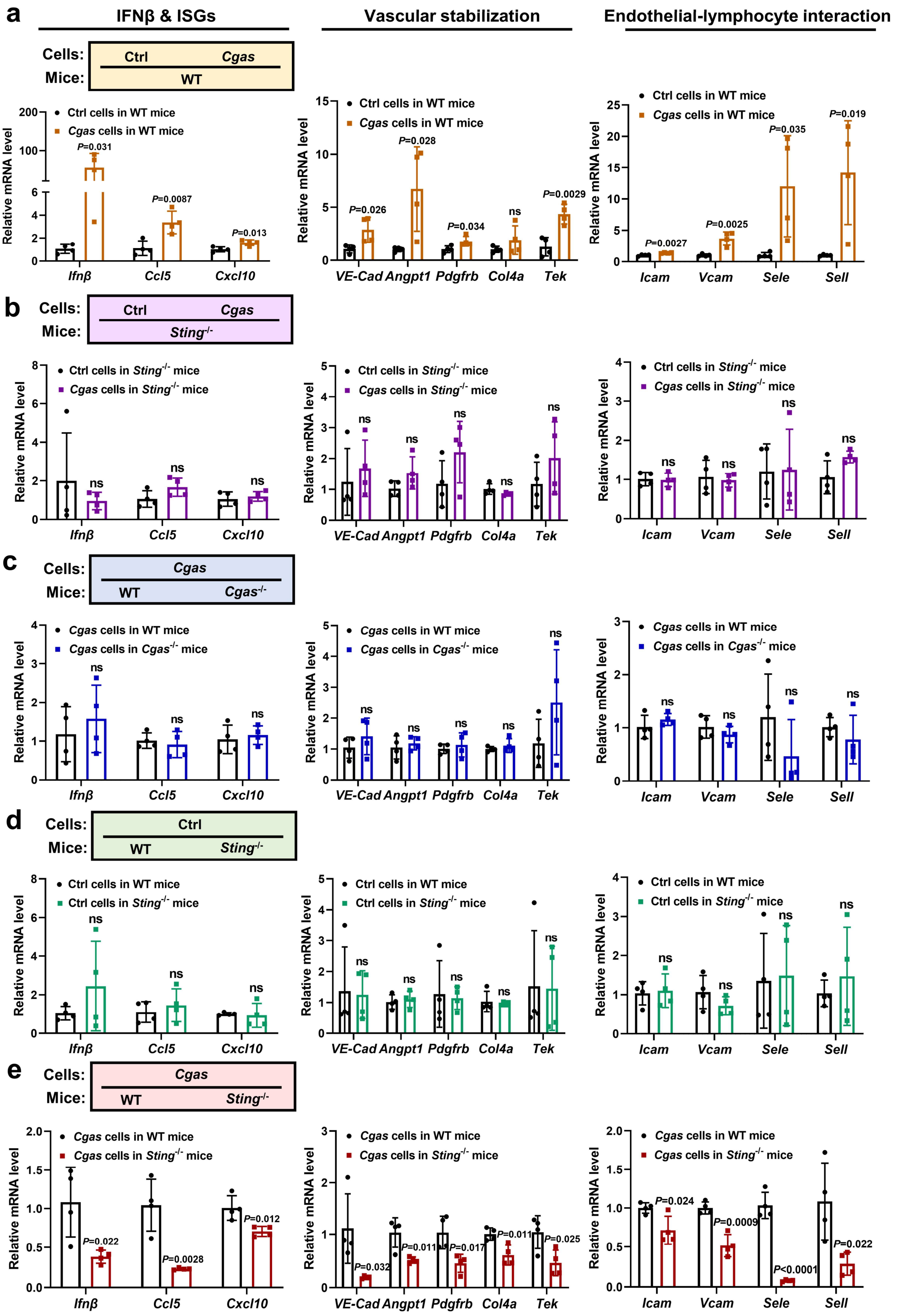

Supplementary Fig. 4 Tumor cGAS and host STING mediates vascular normalizing genes expressions in an interdependence manner. a-e mRNA levels of Ifnβ and ISGs, genes related to vascular stabilization, and endothelial-lymphocyte interaction in indicated tumors (n = 4). *P* values are calculated using two-tailed unpaired Student's *t* test (**a-e**). ns, not significant. Source data are provided as a Source Data file.

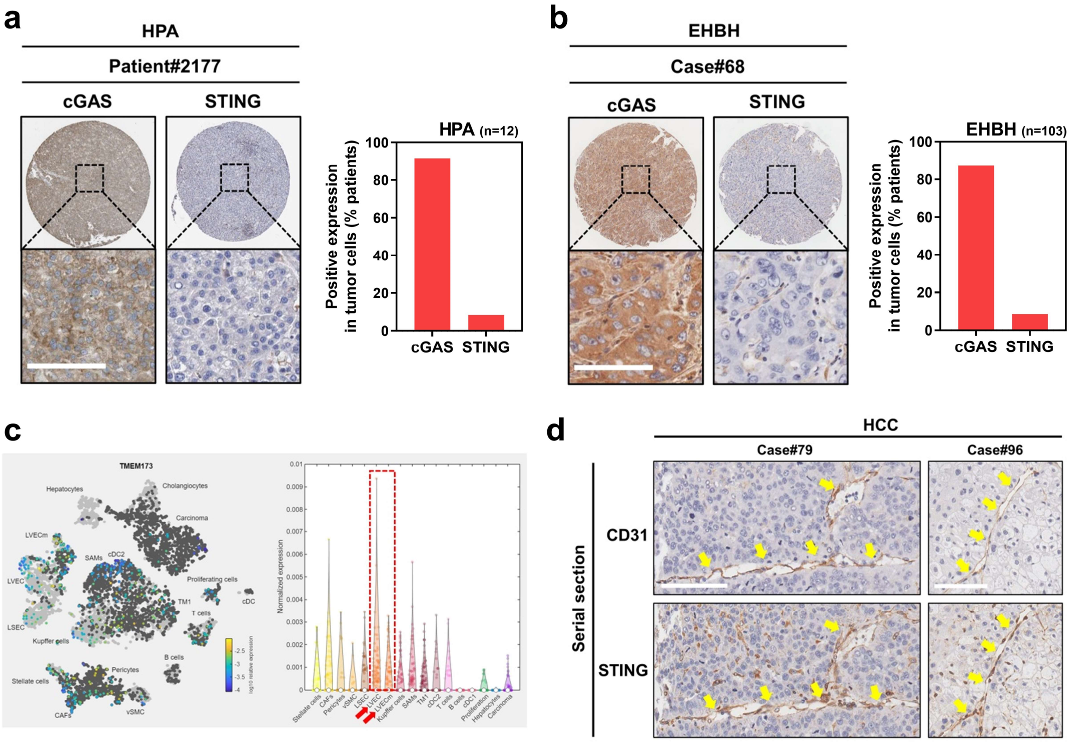

Supplementary Fig. 5 Most liver cancer cells express cGAS, but hardly express STING, whereas endothelial cells show most distinct STING expression in tumor microenvironment. **a**, **b** Representative immunohistochemical images for cGAS and STING expressions in human liver cancer samples from HPA (**a**) and EHBH (**b**) and quantification for the proportion of patients with positive expression of cGAS or STING in liver cancer cells. Scale bars, 100 µm. **c** TMEM173 (STING) mRNA expression in the single cell type clusters identified in malignant and adjacent non-malignant liver tissues from five patients (GEO: GSE146409). The cells formed 17 clusters including hepatocytes, endothelial cells (liver sinusoidal endothelial cells (LSEC), non-tumor liver vascular endothelial cells (LVEC), and tumor liver vascular endothelial cells (LVECm)), mesenchymal cells (stellate cells, cancer-associated fibroblasts (CAFs), pericytes, vascular smooth muscle cells (vSMC)), immune cells (Kupffer cells, scar-associated macrophages (SAMs), tissue monocytes 1 (TM1), cDC1, cDC2, T cells, and B cells), proliferating cells, and carcinoma cells. **d** Representative immunohistochemical images for CD31 and STING expressions in serial sections of HCC samples from the same patient from EHBH. The yellow arrows represent vessels. Scale bars, 100 µm. Source data are provided as a Source Data file.

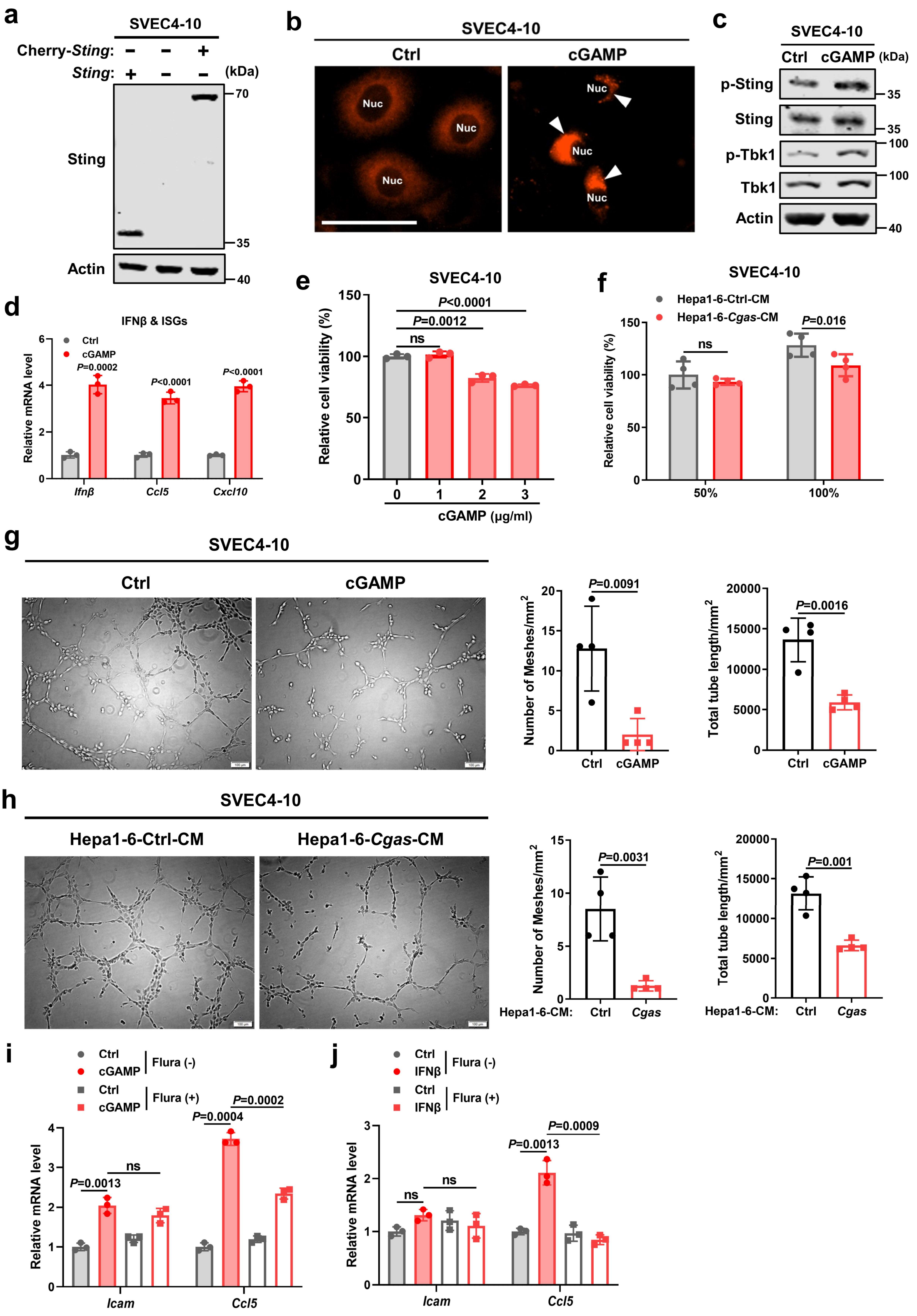

Supplementary Fig. 6 Tumor cell cGAS produces cGAMP to suppress endothelial cell proliferation and angiogenesis. **a** Sting protein levels in SVEC4-10 cells with *Sting*-Cherry overexpression following endogenous Sting knockout. **b** Representative immunofluorescence images for Sting aggregates in SVEC4-10 cells with *Sting*-Cherry overexpression after cGAMP treatment. The white arrows represent Sting aggregates in SVEC4-10 cells. Scale bars, 50 μm. **c**

Protein levels of markers in the Sting pathway including total and p-Sting, total and p-Tbk1 in SVEC4-10 cells after cGAMP treatment. **d** mRNA levels of Ifnβ and ISGs in SVEC4-10 cells after cGAMP treatment (n = 3). **e**, **f** Relative cell viabilities of SVEC4-10 cells treated with Hepa1-6-*Cgas*/Ctrl cells-derived CM (**e**) or different concentrations of cGAMP (**f**) (n = 4). **g**, **h** Representative images and quantification from the Matrigel tube formation assay with SVEC4-10 cell pretreated with Hepa1-6-*Cgas*/Ctrl cells-derived CM (**g**) or cGAMP (**h**) (n = 4). **i** mRNA levels of Icam and Ccl5 in SVEC4-10 cells after cGAMP treatment following Flura pretreatment (n = 3). **j** mRNA levels of Icam and Ccl5 in SVEC4-10 cells after IFNβ treatment following Flura pretreatment (n = 3). Representative of n = 3 independent experiments (**a-c**). *P* values are calculated using two-tailed unpaired Student's *t* test (**d**, **f-h**) and one-way ANOVA (**e**, **i**). ns, not significant. Source data are provided as a Source Data file.

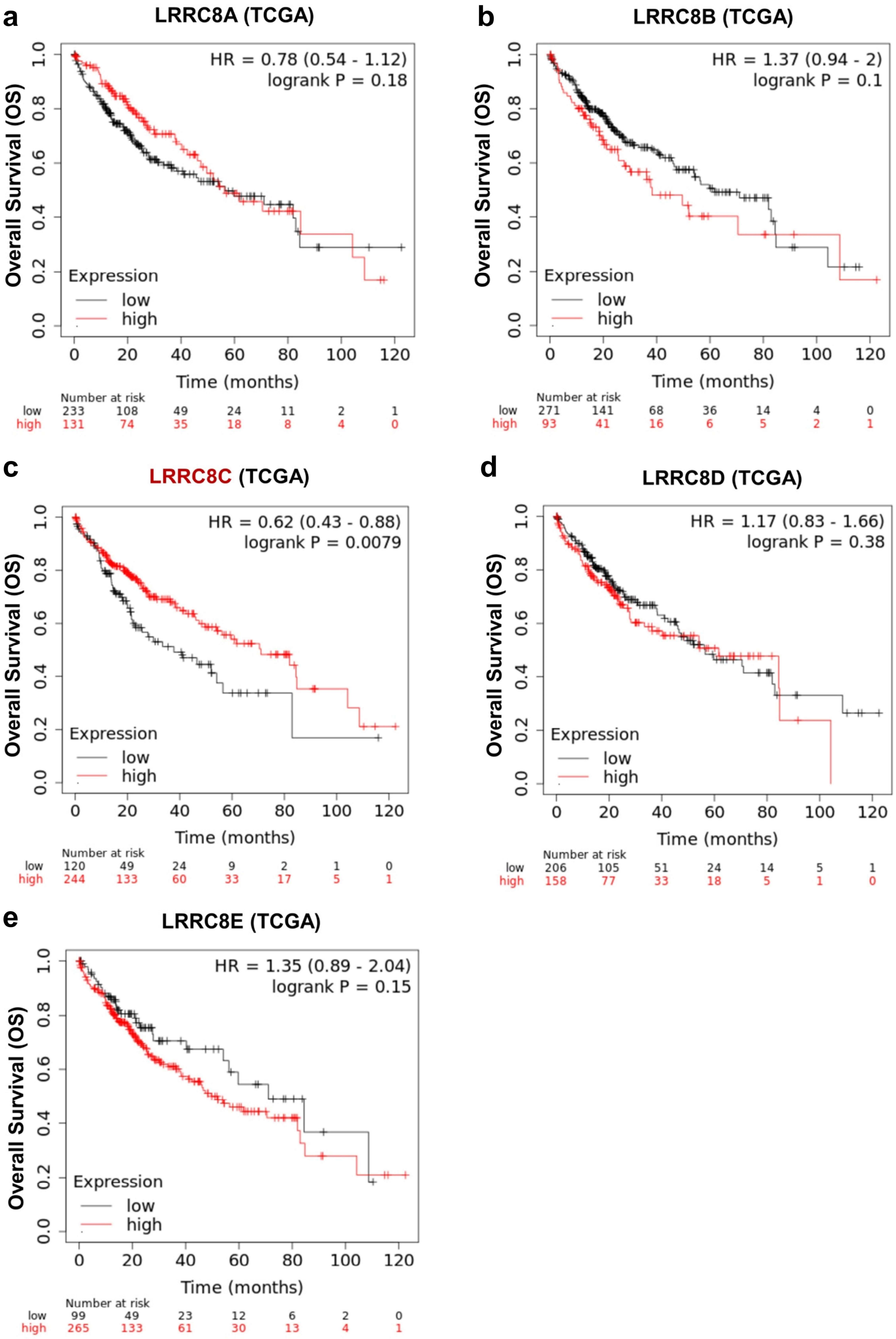

Supplementary Fig. 7 High LRRC8C expression is correlated with better clinical outcomes in liver cancer. a-e Kaplan-Meier analysis of overall survival (OS) in HCC patients according to LRRC8A-E expression from TCGA dataset (n = 364).

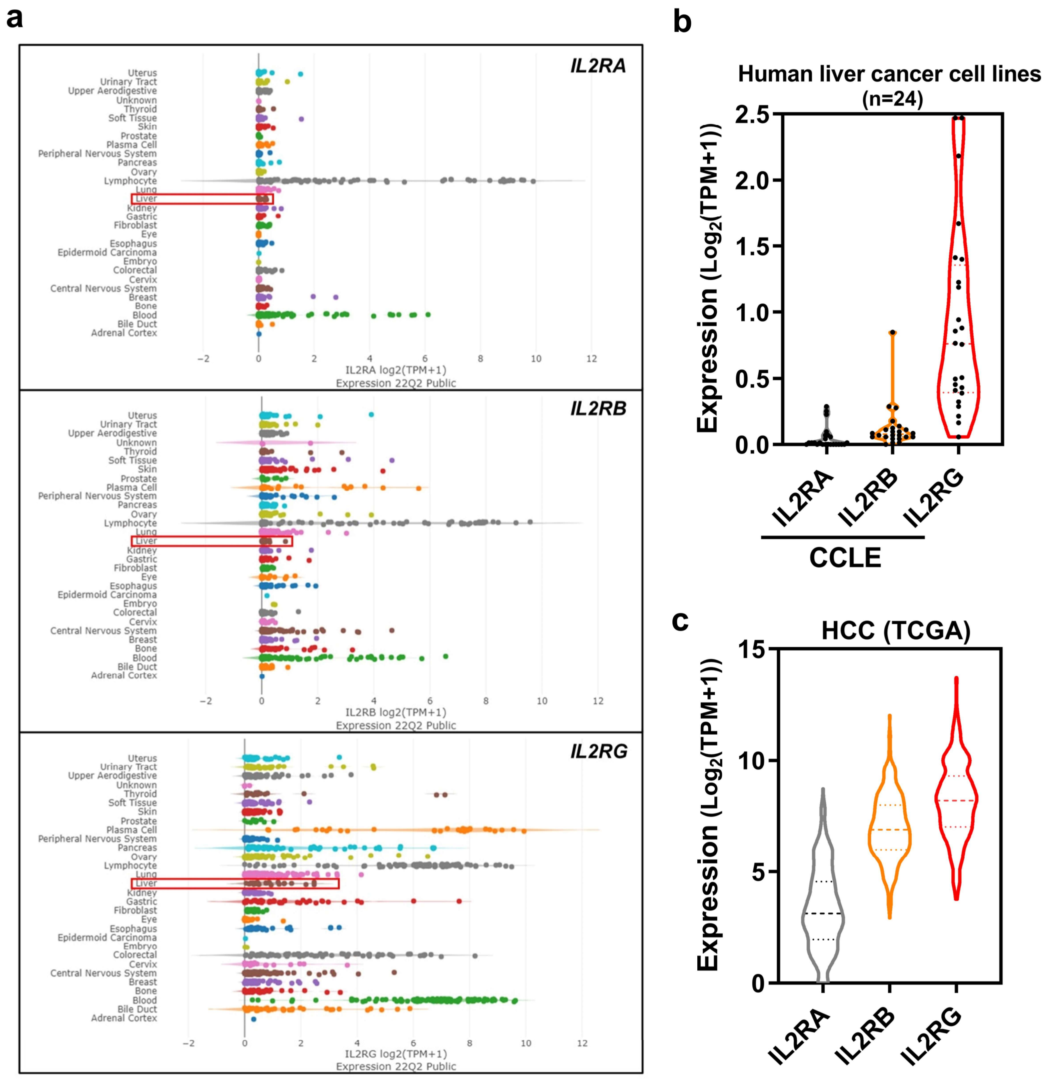

Supplementary Fig. 8 IL2R is also expressed in liver cancer cells. a *IL2RA*, *IL2RB*, and *IL2RG* expressions in cancer cell lines from diverse tissue lineages from CCLE dataset. **b** *IL2RA*, *IL2RB*, and *IL2RG* expressions in liver cancer cell lines from CCLE dataset (n = 24). **c** *IL2RA*, *IL2RB*, and *IL2RG* expressions in HCC from TCGA dataset (n = 371). Source data are provided as a Source Data file.

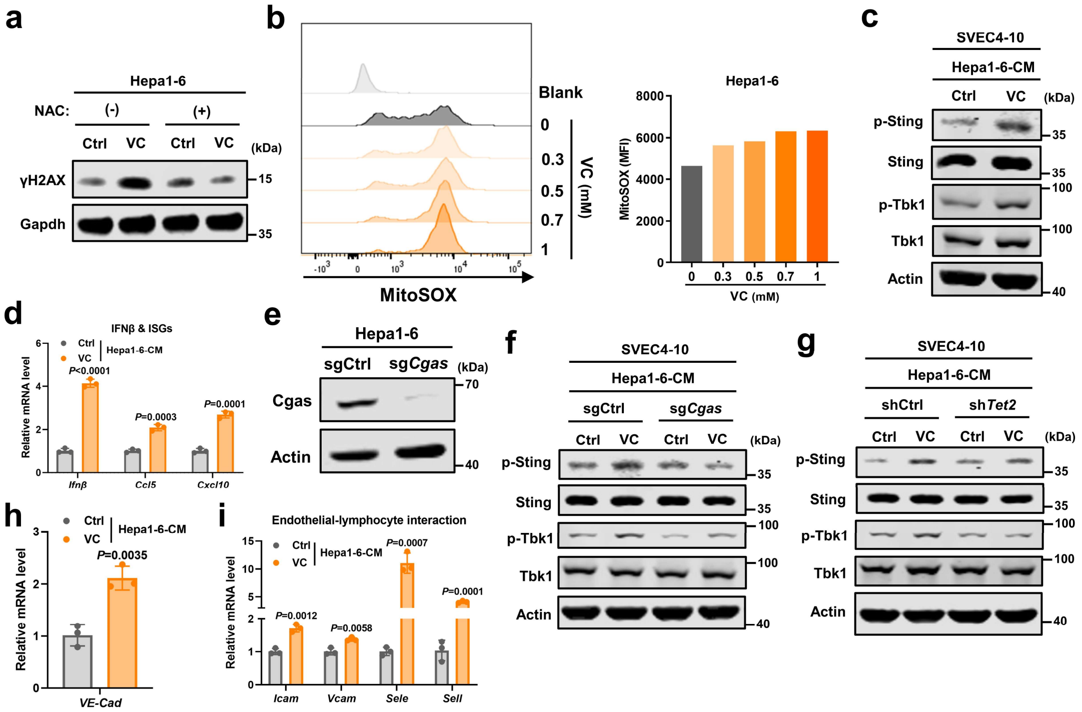

Supplementary Fig. 9 VC-induced TET2 and dsDNA leakage activate tumor cGAS-cGAMP-endothelial STING pathway. **a** γH2AX protein level in Hepa1-6 cells after VC treatment following NAC pretreatment. **b** Flow cytometric analysis and MFI of MitoSOX in Hepa1-6 cells after VC treatment. **c** Protein levels of markers in the Sting pathway including total and p-Sting, total and p-Tbk1 in SVEC4-10 cells after exposure to CM from VC-treated Hepa1-6 cells. **d** mRNA levels of Ifnβ and ISGs in SVEC4-10 cells after exposure to CM from VC-treated Hepa1-6 cells (n = 3). **e** Cgas protein level in Hepa1-6 cells with *Cgas* knocked out by sgRNA (sg*Cgas*) or Ctrl cells (sgCtrl). **f** Protein levels of markers in the Sting pathway including total and p-Sting, total and p-Tbk1 in SVEC4-10 cells after exposure to CM from VC-treated Hepa1-6 sg*Cgas* cells or sgCtrl cells. **g** Protein levels of markers in the Sting pathway including total and p-Sting, total and p-Tbk1 in SVEC4-10 cells after exposure to CM from VC-treated Hepa1-6 sh*Tet2* cells or shCtrl cells. **h** VE-Cad mRNA level in SVEC4-10 cells after exposure to CM from VC-treated Hepa1-6 cells (n = 3). **i** mRNA levels of endothelial-lymphocyte interaction-associated genes in SVEC4-10 cells after exposure to CM from VC-treated Hepa1-6 cells (n = 3). Representative of n = 3 independent experiments (**a**, **c**, **e-g**) and one independent experiment (**b**). *P* values are calculated using two-tailed unpaired Student's *t* test (**d**, **h**, **i**). Source data are provided as a Source Data file.

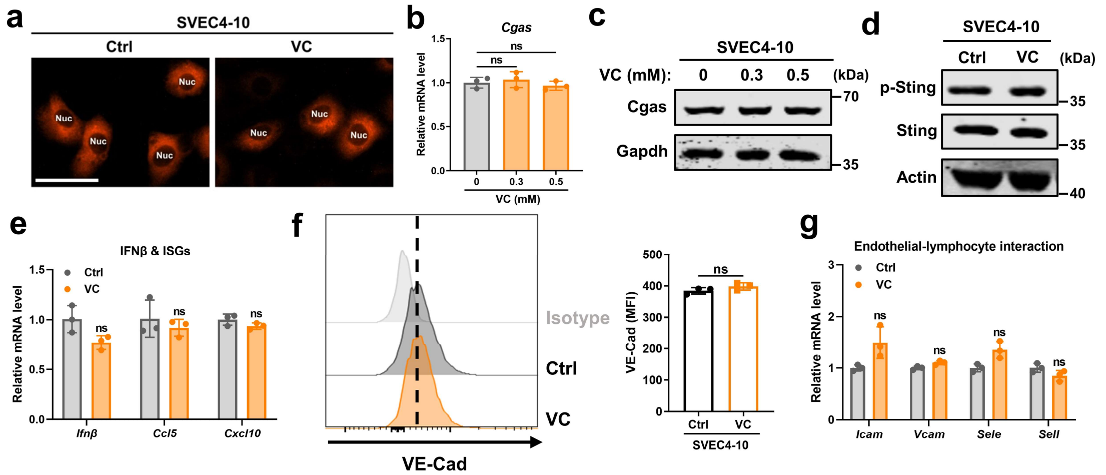

Supplementary Fig. 10 Direct VC stimulation fails to activate endothelial STING pathway. a Representative immunofluorescence images for Sting aggregates in SVEC4-10 cells with *Sting*-Cherry overexpression after VC treatment. Scale bars, 50 μm. **b** *Cgas* mRNA level in SVEC4-10 cells after VC treatment (n = 3). **c** Cgas protein level in SVEC4-10 cells after VC treatment. **d** Protein levels of markers in the Sting pathway including total and p-Sting in SVEC4-10 cells after VC treatment. **e** mRNA levels of Ifnβ and ISGs in SVEC4-10 cells after VC treatment (n = 3). **f** Flow cytometric analysis and MFI of surface VE-Cad in SVEC4-10 cells after VC treatment (n = 3). **g** mRNA levels of endothelial-lymphocyte interaction-associated genes in SVEC4-10 cells after VC treatment (n = 3). Representative of n = 3 independent experiments (**a**, **c**, **d**). *P* values are calculated using one-way ANOVA (**b**) and two-tailed unpaired Student's *t* test (**e-g**). ns, not significant. Source data are provided as a Source Data file.

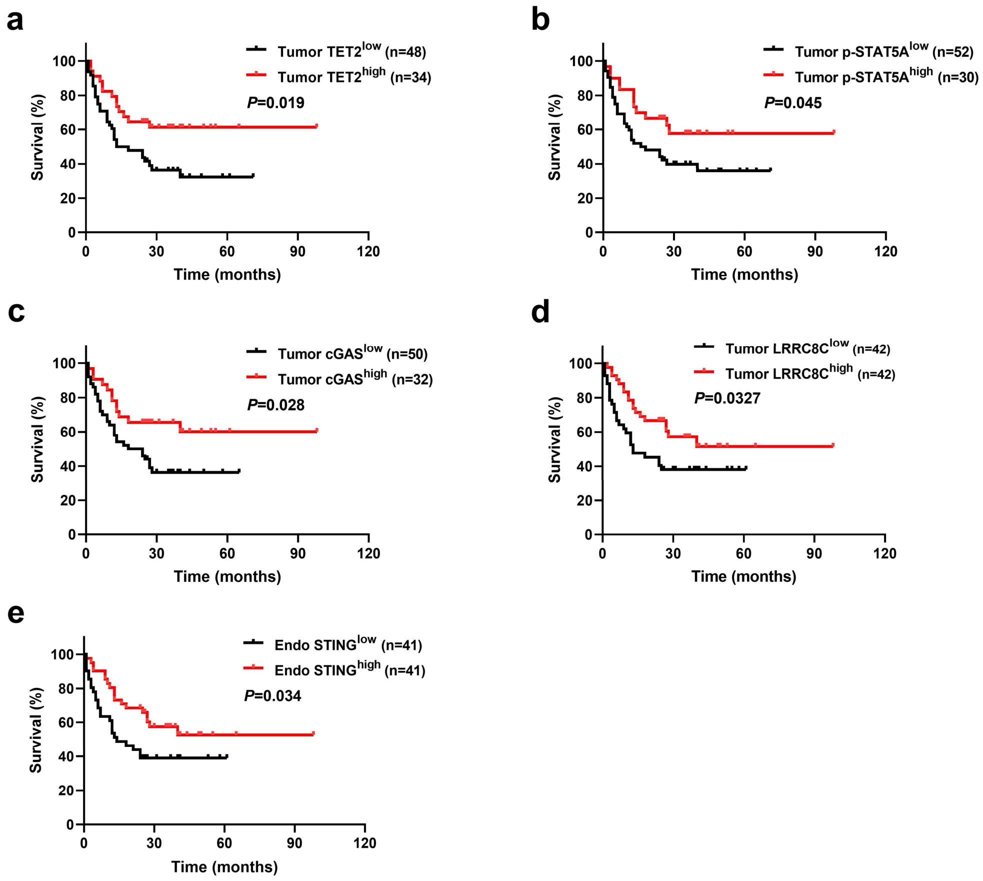

Supplementary Fig. 11 Tumor TET2/p-STAT5A/cGAS/LRRC8C/endothelial STING expressions predict better prognosis in human liver cancer. a-d Kaplan-Meier analysis of disease-free survival (DFS) in HCC patients according to tumor TET2 (**a**), p-STAT5A (**b**), cGAS (**c**), or LRRC8C (**d**) expressions (n = 82). **e** Kaplan-Meier analysis of disease-free survival (DFS) in HCC patients according to endothelial STING expression (n = 82). Source data are provided as a Source Data file.

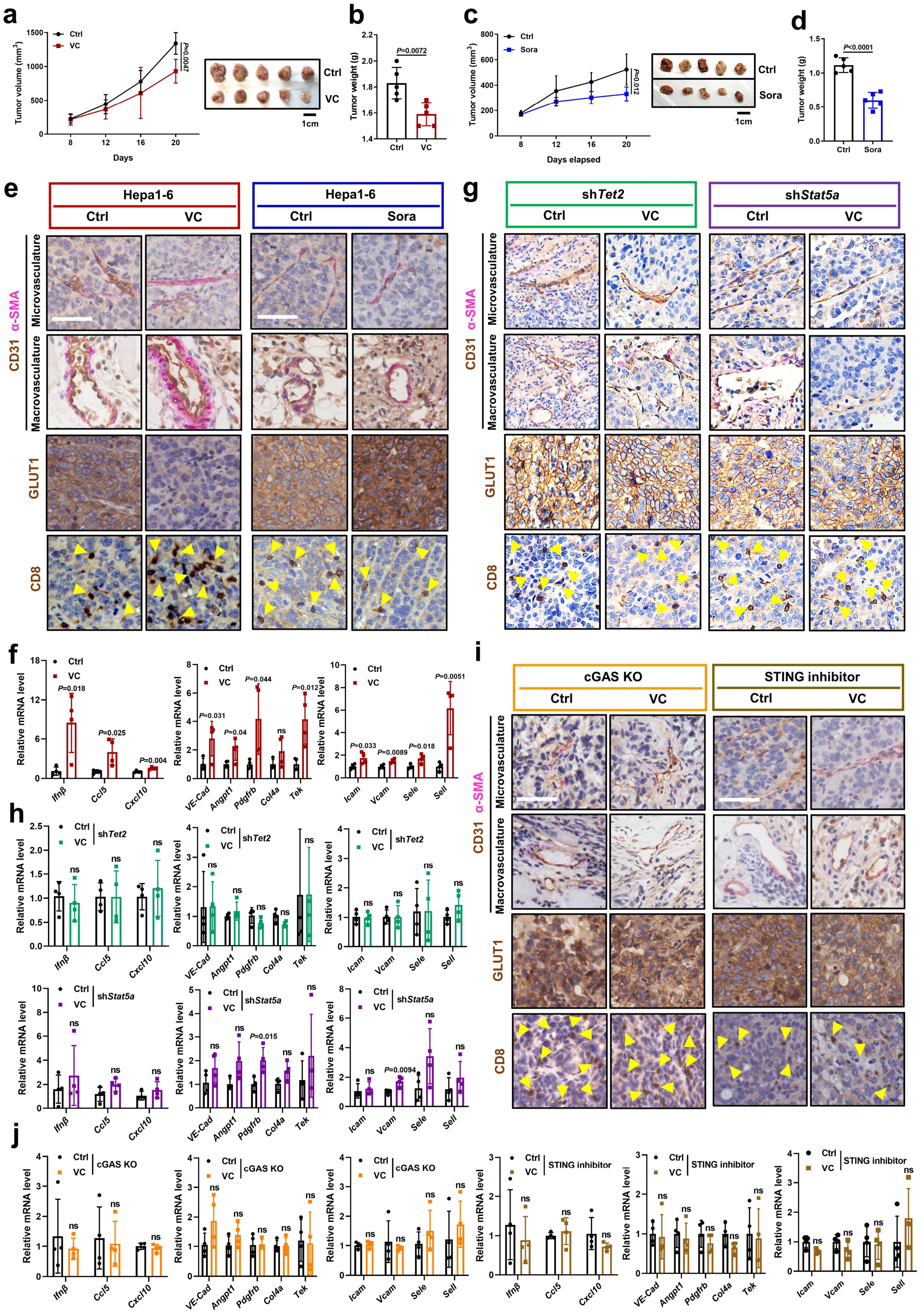

Supplementary Fig. 12 Tumor TET2-STAT5A-cGAS-host STING axis mediates VC-induced vascular normalization. **a**, **b** Tumor growth curves (**a**) and tumor burdens (**b**) in C57BL/6 mice injected subcutaneously with Hepa1-6 cells with VC treatment (n = 5 mice per group). **c**, **d** Tumor growth curves (**c**) and tumor burdens (**d**) in C57BL/6 mice injected subcutaneously with Hepa1-6 cells with Sora treatment (n = 5 mice per group). **e** Representative immunohistochemical images

for $CD31^+$ vessels density, pericyte (α-SMA^+) coverage of vessels ($CD31^+$), $GLUT1^+$ hypoxic area, and $CD8^+$ T cells in VC-treated or Sora-treated tumors (n = 5). The yellow arrows represent $CD8^+$ T cells. Scale bars, 100 µm. **f** mRNA levels of Ifnβ and ISGs, genes related to vascular stabilization, and endothelial-lymphocyte interaction in VC-treated tumors (n = 4). **g** Representative immunohistochemical images for $CD31^+$ vessels density, pericyte (α-SMA^+) coverage of vessels ($CD31^+$), $GLUT1^+$ hypoxic area, and $CD8^+$ T cells in VC-treated sh*Tet2* tumors or VC-treated sh*Stat5a* tumors (n = 5). The yellow arrows represent $CD8^+$ T cells. Scale bars, 100 µm. **h** mRNA levels of Ifnβ and ISGs, genes related to vascular stabilization, and endothelial-lymphocyte interaction in VC-treated sh*Tet2* tumors or VC-treated sh*Stat5a* tumors (n = 4). **i** Representative immunohistochemical images for $CD31^+$ vessels density, pericyte (α-SMA^+) coverage of vessels ($CD31^+$), $GLUT1^+$ hypoxic area, and $CD8^+$ T cells in cGAS KO tumors or STING inhibitor-treated tumors after VC treatment (n = 5). The yellow arrows represent $CD8^+$ T cells. Scale bars, 100 µm. **j** mRNA levels of Ifnβ and ISGs, genes related to vascular stabilization, and endothelial-lymphocyte interaction in cGAS KO tumors or STING inhibitor-treated tumors after VC treatment (n = 4). Data are shown as mean ± SD. *P* values are calculated using two-way ANOVA (**a**, **c**) and two-tailed unpaired Student's *t* test (**b**, **d**, **f**, **h**, **j**). ns, not significant. Source data are provided as a Source Data file.

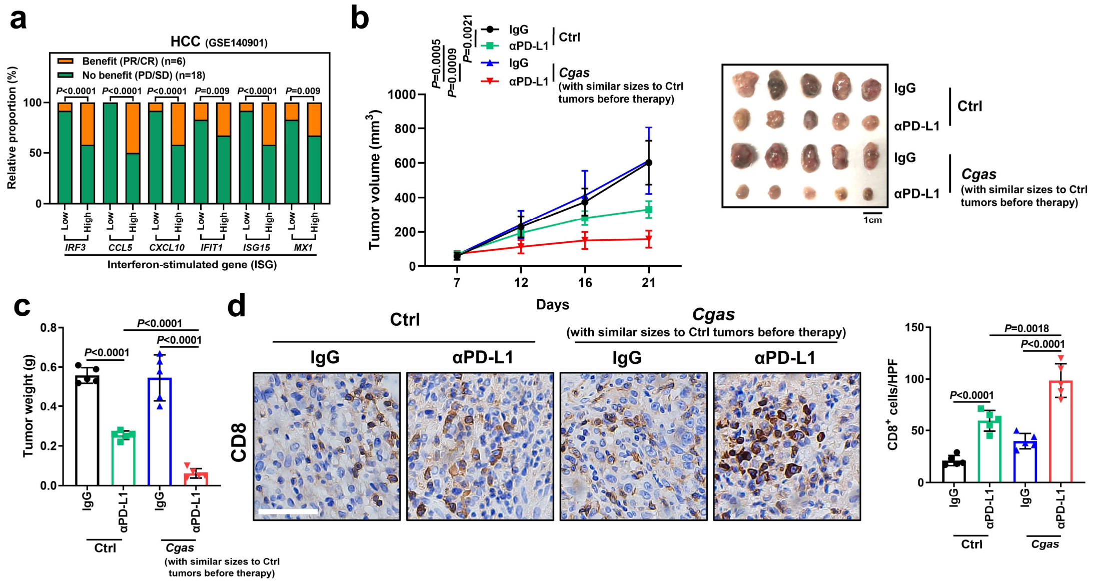

Supplementary Fig. 13 Tumor cGAS potentiates anti-PD-L1 therapy efficacy in liver cancer. **a** Correlation analysis of IFN-stimulated gene (ISG) expression and responses of patients with HCC to anti-PD-1/anti-PD-L1 treatment (n = 24) (GEO: GSE140901). The low and high expression groups were divided relative to the median expression values. PR, partial response; CR, complete response; PD, progressive disease; SD, stable disease. **b**, **c** Tumor growth curves (**b**) and and tumor burdens (**c**) of C57BL/6 mice injected subcutaneously with Ctrl cells and *Cgas* cells (numbers of *Cgas* cells were adjusted to develop similar tumor sizes to Ctrl tumors before therapy) with treatment of αPD-L1or IgG control (n = 5 mice per group). **d** Representative immunohistochemical images and quantification for $CD8^{+}$ T cells in indicated tumors (n = 5). *P* values are calculated using Chi-square test (**a**), two-way ANOVA (**b**), and one-way ANOVA (**c**, **d**). Source data are provided as a Source Data file.

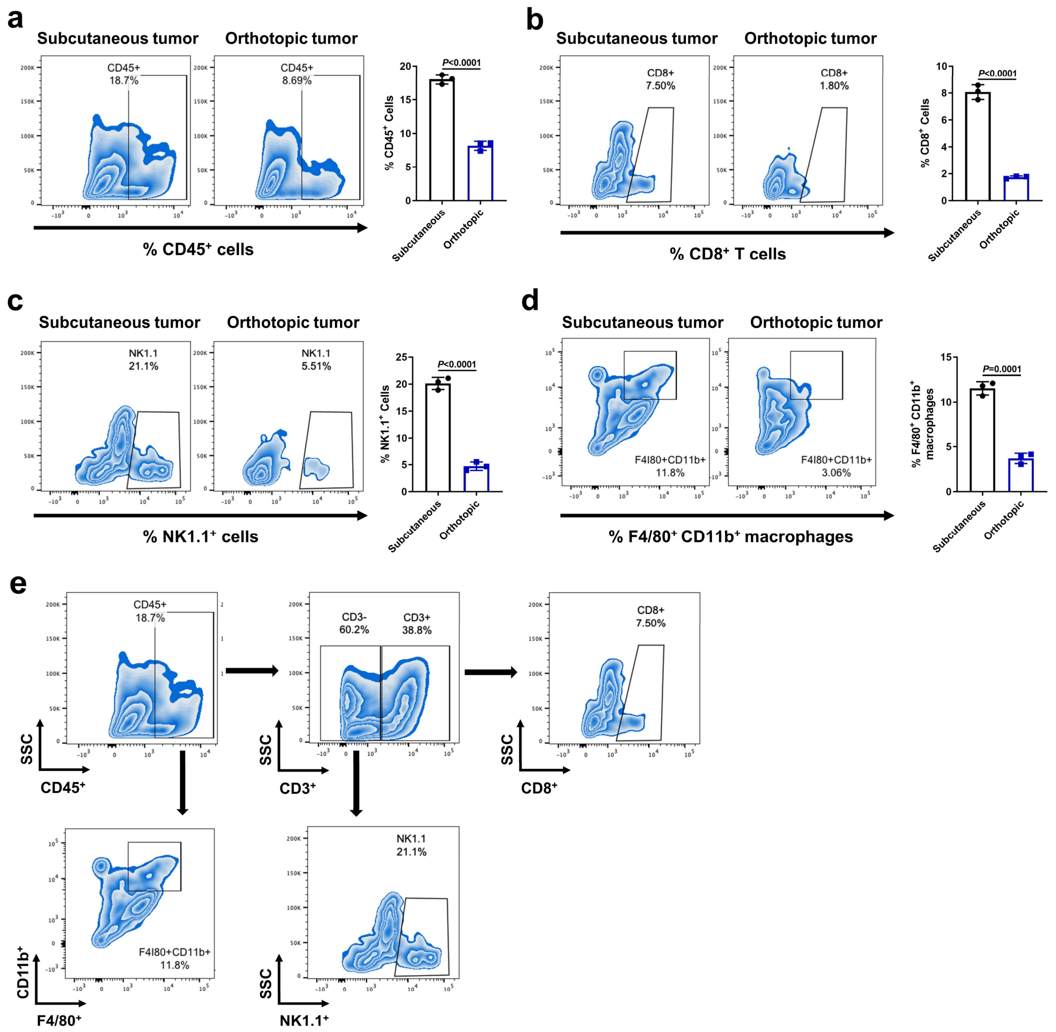

Supplementary Fig. 14 The differences in immune cell infiltration between subcutaneous and orthotopic liver cancer models. a-d Flow cytometric analysis and frequency of CD45^{+} cells (**a**), CD8^{+} T cells (**b**), NK1.1^{+} cells (**c**), and F4/80^{+}CD11b^{+} macrophages (**d**) in subcutaneous tumors versus orthotopic tumors derived from Hepa1-6 cells (n = 3). **e** Flow cytometric gating strategies for indicated cells (Supplementary Fig. 14a, b, Supplementary Fig. 15f). *P* values are calculated using two-tailed unpaired Student's *t* test (**a-d**). Source data are provided as a Source Data file.

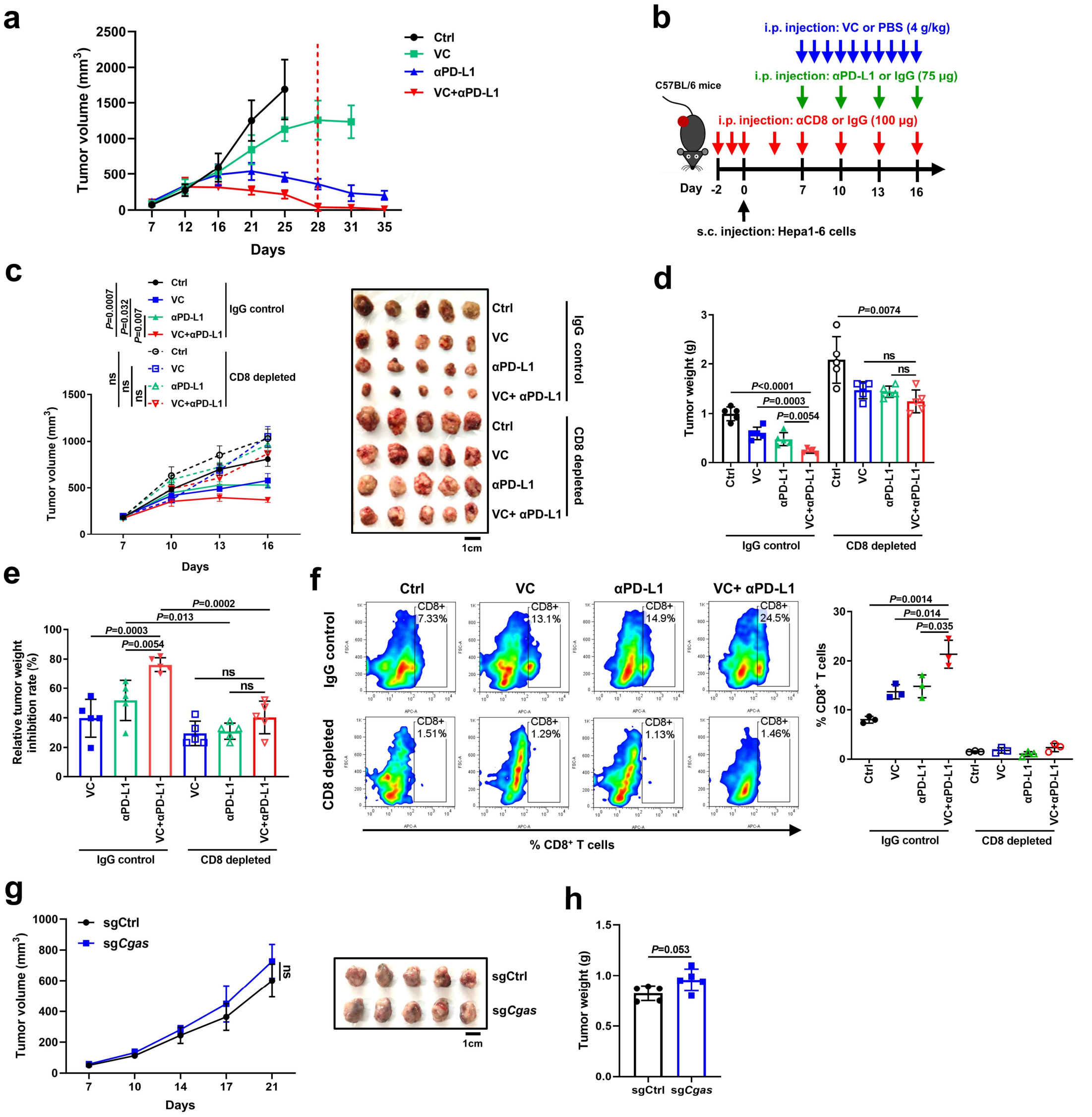

Supplementary Fig. 15 The combinational efficiency of VC and anti-PD-L1 therapy depending on CD8$^+$ T cell-induced anti-tumor immune response. a Tumor growth curves of C57BL/6 mice injected subcutaneously with Hepa1-6 cells with treatment of αPD-L1 and VC either alone or in combination (n = 5 mice per group). **b-d** Scheme representing the experimental procedure (**b**), tumor growth curves (**c**), and tumor burdens (**d**) of C57BL/6 mice injected subcutaneously with Hepa1-6 cells with treatment of αPD-L1 and VC either alone or in combination after CD8 depletion (n = 5 mice per group). **e** Relative tumor weight inhibition rates in tumors with treatment of αPD-L1 and VC either alone or in combination after CD8 depletion (n = 5). **f** Flow cytometric analysis and frequency of CD8$^+$ T cells in tumors with treatment of αPD-L1 and VC either alone or in combination after CD8 depletion (n = 3). **g**, **h** Tumor growth curves (**g**) and tumor burdens (**h**) in C57BL/6 mice injected subcutaneously with Hepa1-6-sg*Cgas* or sgCtrl cells for 3 weeks (n = 5 mice per group). *P* values are calculated using two-way ANOVA (**a**, **c**, **g**), one-way ANOVA (**d-f**), and two-tailed unpaired Student's *t* test (**h**). ns, not significant. Source data are provided as a Source Data file.

Supplementary Table 1. Primers used for qRT-PCR analysis.

Target	Forward primer	Reverse primer
IFNβ (human)	ATGACCAACAAGTGTCTCCTCC	GGAATCCAAGCAAGTTGTAGCTC
CCL5 (human)	CCAGCAGTCGTCTTTGTCAC	CTCTGGGTTGGCACACACTT
CXCL10 (human)	GTGGCATTCAAGGAGTACCTC	TGATGGCCTTCGATTCTGGATT
Ifnβ (mouse)	CAGCTCCAAGAAAGGACGAAC	GGCAGTGTAACTCTTCTGCAT
Ccl5 (mouse)	GCTGCTTTGCCTACCTCTCC	TCGAGTGACAAACACGACTGC
Cxcl10 (mouse)	CCAAGTGCTGCCGTCATTTTC	GGCTCGCAGGGATGATTTCAA
VE-Cad (mouse)	CACTGCTTTGGGAGCCTTC	GGGGCAGCGATTCATTTTTCT
Angpt1 (mouse)	CACATAGGGTGCAGCAACCA	CGTCGTGTTCTGGAAGAATGA
Pdgfrb (mouse)	TTCCAGGAGTGATACCAGCTT	AGGGGGCGTGATGACTAGG
Col4a (mouse)	CTGGCACAAAAGGGACGAG	ACGTGGCCGAGAATTTCACC
Tek (mouse)	GAGTCAGCTTGCTCCTTTATGG	AGACACAAGAGGTAGGGAATTGA
Icam (mouse)	GTGATGCTCAGGTATCCATCCA	CACAGTTCTCAAAGCACAGCG
Vcam (mouse)	AGTTGGGGATTCGGTTGTTCT	CCCCTCATTCCTTACCACCC
Sele (mouse)	ATGCCTCGCGCTTTCTCTC	GTAGTCCCGCTGACAGTATGC
Sell (mouse)	TACATTGCCCAAAAGCCCTTAT	CATCGTTCCATTTCCCAGAGTC
Cgas (mouse)	GAGGCGCGGAAAGTCGTAA	TTGTCCGGTTCCTTCCTGGA
Tet1 (mouse)	ACACAGTGGTGCTAATGCAG	AGCATGAACGGGAGAATCGG
Tet2 (mouse)	AGAGAAGACAATCGAGAAGTCGG	CCTTCCGTACTCCCAAACTCAT
Tet3 (mouse)	TGCGATTGTGTCGAACAAATAGT	TCCATACCGATCCTCCATGAG
Stat1 (mouse)	TCACAGTGGTTCGAGCTTCAG	GCAAACGAGACATCATAGGCA
Stat5a (mouse)	CGCCAGATGCAAGTGTTGTAT	TCCTGGGGATTATCCAAGTCAAT
β-Actin (mouse)	GGCTGTATTCCCCTCCATCG	CCAGTTGGTAACAATGCCATGT
Cgas promoter (mouse)-ChIP	GCAAAATGAGTTCCGCCAAG	TTGGCTGCTGAGATTCCGTA
