# Supplementary material for: TET2-mediated tumor cGAS triggers endothelial STING activation to regulate vasculature remodeling and anti-tumor immunity in liver cancer
Source: Nat Commun. 2024 Jan 4;15:6. doi: 10.1038/s41467-023-43743-9 (PMC10766952; doi:10.1038/s41467-023-43743-9)
Supplement: Supplementary file 3 — Reporting Summary [file 41467_2023_43743_MOESM3_ESM.pdf]

nature portfolio

Corresponding author(s): Wen Yang, Hongyang Wang

Last updated by author(s): Oct 31, 2023

Reporting Summary

Nature Portfolio wishes to improve the reproducibility of the work that we publish. This form provides structure for consistency and transparency in reporting. For further information on Nature Portfolio policies, see our Editorial Policies and the Editorial Policy Checklist.

Statistics

For all statistical analyses, confirm that the following items are present in the figure legend, table legend, main text, or Methods section.

n/a	Confirmed	
☐	☒	The exact sample size (*n*) for each experimental group/condition, given as a discrete number and unit of measurement
☐	☒	A statement on whether measurements were taken from distinct samples or whether the same sample was measured repeatedly
☐	☒	The statistical test(s) used AND whether they are one- or two-sided<br>*Only common tests should be described solely by name; describe more complex techniques in the Methods section.*
☒	☐	A description of all covariates tested
☐	☒	A description of any assumptions or corrections, such as tests of normality and adjustment for multiple comparisons
☐	☒	A full description of the statistical parameters including central tendency (e.g. means) or other basic estimates (e.g. regression coefficient) AND variation (e.g. standard deviation) or associated estimates of uncertainty (e.g. confidence intervals)
☐	☒	For null hypothesis testing, the test statistic (e.g. *F*, *t*, *r*) with confidence intervals, effect sizes, degrees of freedom and *P* value noted<br>*Give P values as exact values whenever suitable.*
☒	☐	For Bayesian analysis, information on the choice of priors and Markov chain Monte Carlo settings
☒	☐	For hierarchical and complex designs, identification of the appropriate level for tests and full reporting of outcomes
☐	☒	Estimates of effect sizes (e.g. Cohen's *d*, Pearson's *r*), indicating how they were calculated

Our web collection on statistics for biologists contains articles on many of the points above.

Software and code

Policy information about availability of computer code

Data collection	IVIS Lumina III (PerkinElmer), ABI PRISM 7300HT Sequence Detection System (Applied Biosystems), LSRFortessa flow cytometer (BD Biosciences),STELLARIS 5 confocal microscope (Leica Microsystems),Odyssey fluorescence scanner (Li-Cor, Lincoln, NE, USA).
Data analysis	Statistical analysis was performed using GraphPad Prism 8 software (v8.4.2.679, GraphPad Software, Inc., San Diego CA, USA).

For manuscripts utilizing custom algorithms or software that are central to the research but not yet described in published literature, software must be made available to editors and reviewers. We strongly encourage code deposition in a community repository (e.g. GitHub). See the Nature Portfolio guidelines for submitting code & software for further information.

Data

Policy information about availability of data

All manuscripts must include a data availability statement. This statement should provide the following information, where applicable:

- Accession codes, unique identifiers, or web links for publicly available datasets
- A description of any restrictions on data availability
- For clinical datasets or third party data, please ensure that the statement adheres to our policy

Publicly available datasets reported in this paper are from the GEO databases (GSE51401, GSE69164, GSE146409, and GSE140901), The Cancer Genome Atlas (TCGA), the International Cancer Genome Consortium (ICGC), the Human Protein Atlas (HPA), and the Cancer Cell Line Encyclopedia (CCLE). The remaining data are available within the Article, Supplementary Information or Source Data file. Source data are provided with this paper.

Research involving human participants, their data, or biological material

Policy information about studies with human participants or human data. See also policy information about sex, gender (identity/presentation), and sexual orientation and race, ethnicity and racism.

Reporting on sex and gender	Tumor tissues from patients with HCC were obtained from the Eastern Hepatobiliary Surgery Hospital (EHBH), Shanghai, China, , with male to female ratio of 6:1, conforming to the sex disparities in HCC, which has a strong male predominance.
Reporting on race, ethnicity, or other socially relevant groupings	*Please specify the socially constructed or socially relevant categorization variable(s) used in your manuscript and explain why they were used. Please note that such variables should not be used as proxies for other socially constructed/relevant variables (for example, race or ethnicity should not be used as a proxy for socioeconomic status).* *Provide clear definitions of the relevant terms used, how they were provided (by the participants/respondents, the researchers, or third parties), and the method(s) used to classify people into the different categories (e.g. self-report, census or administrative data, social media data, etc.)* *Please provide details about how you controlled for confounding variables in your analyses.*
Population characteristics	Tumor tissues from patients with HCC were obtained from the Eastern Hepatobiliary Surgery Hospital (EHBH) (Shanghai, China,) with median age of 50.
Recruitment	No patients were recruited specifically for this study.
Ethics oversight	Patient consent was obtained prior to the commencement of the study. All procedures performed in the study were approved by the Ethical Committee of the Second Military Medical University and in accordance with the Declaration of Helsinki.

Note that full information on the approval of the study protocol must also be provided in the manuscript.

Field-specific reporting

Please select the one below that is the best fit for your research. If you are not sure, read the appropriate sections before making your selection.

☒ Life sciences ☐ Behavioural & social sciences ☐ Ecological, evolutionary & environmental sciences

For a reference copy of the document with all sections, see nature.com/documents/nr-reporting-summary-flat.pdf

Life sciences study design

All studies must disclose on these points even when the disclosure is negative.

Sample size	Although we did not use statistical methods to calculate sample size, we used a minimum of 3 biological replicates. For mice experiments, we based numbers on our previously published experiments (PMID: 33171124), taking into account the animal welfare.
Data exclusions	No data were excluded.
Replication	Experimental findings were reliably reproduced. The number (n) of biological replicates or animals is indicated as an exact number in the figure legends.
Randomization	Mice were randomly assigned to different treatment groups.
Blinding	Blinding was not performed in cell and mouse experiments. Because the investigator had to know the groups to perform the study. The experimenters were blinded for the analysis of the histopathological scores for experimental groups.

Reporting for specific materials, systems and methods

We require information from authors about some types of materials, experimental systems and methods used in many studies. Here, indicate whether each material, system or method listed is relevant to your study. If you are not sure if a list item applies to your research, read the appropriate section before selecting a response.

Materials & experimental systems

n/a	Involved in the study
☐	☒ Antibodies
☐	☒ Eukaryotic cell lines
☒	☐ Palaeontology and archaeology
☐	☒ Animals and other organisms
☒	☐ Clinical data
☒	☐ Dual use research of concern
☒	☐ Plants

Methods

n/a	Involved in the study
☒	☐ ChIP-seq
☐	☒ Flow cytometry
☒	☐ MRI-based neuroimaging

Antibodies

Antibodies used

Anti-human cGAS (79978, 1:1000), anti-mouse cGAS (31659, 1:1000), anti-human/mouse STING (13647, 1:1000), anti-mouse p-STING (Ser365) (72971, 1:1000), anti-human/mouse TBK1 (38066, 1:1000), anti-human/mouse p-TBK1(Ser172) (5483, 1:1000), anti-human/mouse α-SMA (19245, 1:100) antibodies, and anti-human/mouse γH2AX (9718, 1:1000) were purchased from Cell Signaling Technology. Anti-human/mouse LRRC8C (21601-1-AP, 1:1000), anti-human/mouse Flag (20543-1-AP, 1:1000), anti-human/mouse GAPDH (60004-1-Ig, 1:10000), and anti-human/mouse β-Actin (66009-1-Ig, 1:10000) were purchased from Proteintech. Anti-human/mouse TET2 (GTX124205, 1:1000) and anti-human/mouse p-STAT5A (GTX13593, 1:1000) were purchased from GeneTex. Anti-human/mouse CD31 (ab182981, 1:100), anti-human/mouse GLUT1 (ab115730, 1:500), anti-human CD8 (ab93278, 1:100), anti-mouse CD8 (ab209775, 1:100), anti-dsDNA (ab27156, 1:500), anti-mouse NKp46 (ab233558, 1:200), and anti-human/mouse HSP60 (ab46798, 1:200) were purchased from Abcam. Anti-human/mouse VEGFR2 (sc-6251, 1:1000) was purchased from Santa Cruz. Anti-mouse VE-Cad (CD144) (138011, 1:100), anti-mouse CD45 (103112/103108, 1:100), anti-mouse CD3 (100214, 1:100), anti-mouse CD8 (100706, 1:100), anti-mouse NK1.1 (108714, 1:100), anti-mouse CD11b (101224, 1:100), and anti-mouse F4/80 (123116, 1:100) antibodies were purchased from Biolegend. InVivoMab anti-mouse PD-L1 (BE0101), anti-mouse CD8 (BE0004), and IgG2a isotype (BE0089) antibodies were purchased from BioXCell.

Validation

Antibody (dilution, Company, Cat number):

anti-human cGAS (1:1000, Cell Signaling Technology,79978), https://www.cellsignal.cn/products/primary-antibodies/cgas-e5v3w-rabbit-mab/79978?site-search-type=Products&N=4294956287&Ntt=79978&fromPage=plp&_requestid=1208386

anti-mouse cGAS (1:1000, Cell Signaling Technology,31659), https://www.cellsignal.cn/products/primary-antibodies/cgas-d3o8o-rabbit-mab/31659?site-search-type=Products&N=4294956287&Ntt=31659&fromPage=plp&_requestid=1208808

anti-human/mouse STING (1:1000, Cell Signaling Technology,13647), https://www.cellsignal.cn/products/primary-antibodies/sting-d2p2f-rabbit-mab/13647?site-search-type=Products&N=4294956287&Ntt=13647&fromPage=plp&_requestid=1208855

anti-mouse p-STING (Ser365) (1:1000, Cell Signaling Technology,72971), https://www.cellsignal.cn/products/primary-antibodies/phospho-sting-ser365-d8f4w-rabbit-mab/72971?site-search-type=Products&N=4294956287&Ntt=72971&fromPage=plp&_requestid=1208945

anti-human/mouse TBK1 (1:1000, Cell Signaling Technology,38066), https://www.cellsignal.cn/products/primary-antibodies/tbk1-nak-e8i3g-rabbit-mab/38066?site-search-type=Products&N=4294956287&Ntt=38066&fromPage=plp&_requestid=1208993

anti-human/mouse p-TBK1(Ser172) (1:1000, Cell Signaling Technology,5483), https://www.cellsignal.cn/products/primary-antibodies/phospho-tbk1-nak-ser172-d52c2-xp-rabbit-mab/5483?site-search-type=Products&N=4294956287&Ntt=5483&fromPage=plp&_requestid=1220418

anti-human/mouse α-SMA (1:100, Cell Signaling Technology,19245), https://www.cellsignal.cn/products/primary-antibodies/a-smooth-muscle-actin-d4k9n-xp-rabbit-mab/19245?site-search type=Products&N=4294956287&Ntt=19245&fromPage=plp&_requestid=1220569

anti-human/mouse γH2AX (1:1000, Cell Signaling Technology,9718) https://www.cellsignal.cn/products/primary-antibodies/phospho-histone-h2a-x-ser139-20e3-rabbit-mab/9718?site-search-type=Products&N=4294956287&Ntt=9718&fromPage=plp&_requestid=1220659

Anti-human/mouse LRRC8C (1:1000, Proteintech, 21601-1-AP), https://www.ptgcn.com/products/LRRC8C-Antibody-21601-1-AP.htm

anti-human/mouse Flag (1:1000, Proteintech, 20543-1-AP), https://www.ptgcn.com/products/Flag-Tag-Antibody-20543-1-AP.htm

anti-human/mouse GAPDH (1:10000, Proteintech, 60004-1-Ig), https://www.ptgcn.com/products/GAPDH-Antibody-60004-1-Ig.htm

and anti-human/mouse β-Actin (1:10000, Proteintech, 66009-1-Ig), https://www.ptgcn.com/products/Pan-Actin-Antibody-66009-1-Ig.htm

Anti-human/mouse TET2 (1:1000, GeneTex, GTX124205), https://www.genetex.cn/Product/Detail/TET2-antibody-N2-2-N-term/GTX124205

anti-human/mouse p-STAT5A (1:1000, GeneTex, GTX13593), https://www.genetex.cn/Product/Detail/STAT5A-phospho-Tyr694-antibody/GTX13593

anti-human/mouse CD31 (1:100, Abcam, ab182981), https://www.abcam.cn/products/primary-antibodies/cd31-antibody-epr17259-ab182981.html

anti-human/mouse GLUT1 (1:500, Abcam, ab115730), https://www.abcam.cn/products/primary-antibodies/glucose-transporter-glut1-antibody-epr3915-ab115730.html

anti-human CD8 (1:100, Abcam, ab93278), https://www.abcam.cn/products/primary-antibodies/cd8-alpha-antibody-ep1150y-ab93278.html

anti-mouse CD8 (1:100, Abcam, ab209775), https://www.abcam.cn/products/primary-antibodies/cd8-alpha-antibody-epr20305-ab209775.html

anti-dsDNA (1:500, Abcam, ab27156), https://www.abcam.cn/products/primary-antibodies/ds-dna-antibody-35i9-dna-bsa-and-azide-free-ab27156.html

anti-mouse NKp46 (1:200, Abcam, ab233558), https://www.abcam.cn/products/primary-antibodies/ncr1-antibody-epr23097-35-ab233558.html

anti-human/mouse HSP60 (1:200, Abcam, ab46798), https://www.abcam.cn/products/primary-antibodies/hsp60-antibody-ab46798.html

Anti-human/mouse VEGFR2 (1:1000, Santa Cruz, sc-6251), https://www.scbt.com/p/vegfr2-antibody-a-3?requestFrom=search

Anti-mouse VE-Cad (CD144) (1:100, Biolegend, 138011), https://www.biolegend.com/en-us/products/apc-anti-mouse-cd144-ve-cadherin-antibody-6989

Anti-mouse CD45 (1:100, Biolegend, 103112), https://www.biolegend.com/en-us/products/apc-anti-mouse-cd45-antibody-97

Anti-mouse CD45 (1:100, Biolegend, 103108), https://www.biolegend.com/en-us/products/fitc-anti-mouse-cd45-antibody-99

anti-mouse CD3 (1:100, Biolegend, 100214), https://www.biolegend.com/en-us/products/pacific-blue-anti-mouse-cd3-antibody-3317

anti-mouse CD8 (1:100, Biolegend, 100706), https://www.biolegend.com/en-us/products/fitc-anti-mouse-cd8a-antibody-153

anti-mouse NK1.1 (1:100, Biolegend, 108714), https://www.biolegend.com/en-us/products/pe-cyanine7-anti-mouse-nk-1-1-antibody-2840

anti-mouse CD11b (1:100, Biolegend, 101224), https://www.biolegend.com/en-us/products/pacific-blue-anti-mouse-human-cd11b-antibody-3863

anti-mouse F4/80 (1:100, Biolegend, 123116), https://www.biolegend.com/en-us/products/apc-anti-mouse-f4-80-antibody-4071

InVivoMab anti-mouse PD-L1 (BioXCell, BE0101), https://www.bioxcell.com.cn/in-vivo-antibodies/m-pdl-1.html

anti-mouse CD8 (BioXCell, BE0004), https://www.bioxcell.com.cn/in-vivo-antibodies/m-cd8a.html

IgG2a isotype (BioXCell, BE0089), https://www.bioxcell.com.cn/product/catalogsearch.html?q=BE0089

Eukaryotic cell lines

Policy information about cell lines and Sex and Gender in Research

Cell line source(s)	The murine liver cancer cell line Hepa1-6 (SCSP-512) and human HCC cell lines Huh7 (SCSP-526) were purchased from Cell Bank of Type Culture Collection of Chinese Academy of Sciences (CBTCCCAS, Shanghai, China). The murine endothelial cell line SVEC4-10 (CRL-2181) was purchased from American Type Culture Collection (ATCC; Manassas, VA, USA).
Authentication	Cell lines were authenticated by STR profiling.
Mycoplasma contamination	Cell lines were verified to be mycoplasma negative.
Commonly misidentified lines (See ICLAC register)	none

Animals and other research organisms

Policy information about studies involving animals; ARRIVE guidelines recommended for reporting animal research, and Sex and Gender in Research

Laboratory animals	Cgas knockout mice (Cgas-/-) (stock number: 026554) and Sting knockout mice (Sting-/-) (stock number: 025805), both on C57BL/6 background, were purchased from Jackson Laboratory. C57BL/6 mice were used as wild-type (WT) mice in this study and obtained from Laboratory Animal Resources, Chinese Academy of Sciences (Shanghai, China). Male mice at 5-6 weeks of age used in the

	experiments were obtained from the GemPharmatech Co., Ltd (Jiangsu, China). Mice were housed under 12 light/12 dark cycle, temperatures of 22±2 ℃ with 50±10 % humidity.
Wild animals	No wild animals were used.
Reporting on sex	Male animals were used because HCC has a strong male predominance.
Field-collected samples	No field-collected samples were used.
Ethics oversight	All animal protocols used in this study were approved by The University Committee on Use and Care of Animals of Second Military Medical University.

Note that full information on the approval of the study protocol must also be provided in the manuscript.

Plants

Seed stocks	*Report on the source of all seed stocks or other plant material used. If applicable, state the seed stock centre and catalogue number. If plant specimens were collected from the field, describe the collection location, date and sampling procedures.*
Novel plant genotypes	*Describe the methods by which all novel plant genotypes were produced. This includes those generated by transgenic approaches, gene editing, chemical/radiation-based mutagenesis and hybridization. For transgenic lines, describe the transformation method, the number of independent lines analyzed and the generation upon which experiments were performed. For gene-edited lines, describe the editor used, the endogenous sequence targeted for editing, the targeting guide RNA sequence (if applicable) and how the editor was applied.*
Authentication	*Describe any authentication procedures for each seed stock used or novel genotype generated. Describe any experiments used to assess the effect of a mutation and, where applicable, how potential secondary effects (e.g. second site T-DNA insertions, mosiacism, off-target gene editing) were examined.*

Flow Cytometry

Plots

Confirm that:

☒ The axis labels state the marker and fluorochrome used (e.g. CD4-FITC).

☒ The axis scales are clearly visible. Include numbers along axes only for bottom left plot of group (a 'group' is an analysis of identical markers).

☒ All plots are contour plots with outliers or pseudocolor plots.

☒ A numerical value for number of cells or percentage (with statistics) is provided.

Methodology

Sample preparation	Mice tumors were mechanically minced and incubated in collagenase IV (2 mg/mL, Sigma) and DNase I (50 µg/mL, Sigma) for 30 min at 37°C with shaking. The dissociated cells were filtered through a 70 µm cell strainer (BD). Then, the resulting single cell suspensions were incubated with Fc block and stained with the indicated surface antibodies for 20 min at 37°C.
Instrument	Cells were performed on LSRFortessa X-20 flow cytometer (BD Bioscience).
Software	Data analysis was performed using FlowJo V10.
Cell population abundance	At least 5,000 cells were analyzed for each sample.
Gating strategy	CD3+ T cells: CD45+CD3+; CD8+ T cells:CD45+CD3+CD8+; NK cells: CD45+CD3-NK1,1+; Macrophages: CD45+CD11b+F4/80+.

☒ Tick this box to confirm that a figure exemplifying the gating strategy is provided in the Supplementary Information.
